# The impact of serum 25-hydroxyvitamin D, calcium, and parathyroid hormone levels on the risk of coronary artery disease in patients with diabetes: a Mendelian randomization study

**DOI:** 10.1186/s12937-021-00735-z

**Published:** 2021-10-03

**Authors:** Songzan Chen, Fangkun Yang, Tian Xu, Yao Wang, Kaijie Zhang, Guosheng Fu, Wenbin Zhang

**Affiliations:** 1 Department of Cardiology, Key Laboratory of Cardiovascular Intervention and Regenerative Medicine of Zhejiang Province, Sir Run Run Shaw Hospital, School of Medicine, Zhejiang University, 3 East Qingchun Road, Hangzhou, 310016 Zhejiang Province China; 2 Department of Cardiology, Ningbo First Hospital, School of Medicine, Zhejiang University, Ningbo, 315010 China

**Keywords:** Causal association, Coronary artery disease, Diabetes, Mendelian randomization, Serum 25-hydroxyvitamin D levels, Serum calcium levels, Serum parathyroid hormone levels

## Abstract

**Background:**

To investigate the causal association between serum 25-hydroxyvitamin D (25OHD), calcium (Ca), and parathyroid hormone (PTH) levels and the risk of coronary artery disease (CAD) in patients with diabetes using a Mendelian randomization approach.

**Methods:**

Genetic signatures associated with serum 25OHD, Ca, and PTH levels were extracted from recently published genome-wide association study (GWAS), including 79,366, 39,400, 29,155 individuals, respectively. Genetic association estimates for CAD in patients with diabetes were obtained from a GWAS of 15,666 individuals with diabetes (3,968 CAD cases, 11,696 controls). The inverse-variance-weighted method was employed for the primary analysis, and other robust methods were applied for sensitivity analyses.

**Results:**

Six, seven and five single nucleotide polymorphisms were identified as instrumental variables for serum 25OHD, Ca and PTH levels, respectively. There was no significant association between genetically predicted serum 25OHD levels and the risk of CAD in patients with diabetes (odds ratio (OR) = 1.04, 95% confidence interval (CI): 0.58 - 1.87, *P* = 0.888). Similarly, genetically predicted serum Ca (OR = 1.83, 95% CI: 0.62 – 5.35, *P* = 0.273) and PTH levels (OR = 1.27, 95% CI: 0.67 – 2.44, *P* = 0.464) were not significantly associated with the risk of CAD in patients with diabetes. These findings were robust in sensitivity analyses.

**Conclusions/interpretation:**

Serum 25OHD, Ca and PTH levels may not be causally associated with the risk of CAD in patients with diabetes.

**Supplementary Information:**

The online version contains supplementary material available at 10.1186/s12937-021-00735-z.

## Research in context

### What is already known about this subject?

- A recent Mendelian randomization study and several observational studies indicated that elevated serum calcium levels were related to increased risk of coronary artery disease in general population.
- Serum 25-hydroxyvitamin D and parathyroid hormone levels were suggested to be related to coronary artery disease in general population, however, Mendelian randomization studies and randomized controlled trials indicated no significant association.

### What is the key question?

- Dose genetically predicted serum 25-hydroxyvitamin D, calcium and parathyroid hormone levels causally affect the risk of coronary artery disease in patients with diabetes?

### What are the new findings?

- Genetically predicted serum 25-hydroxyvitamin D, calcium and parathyroid hormone levels may not be causally associated with the risk of coronary artery disease in patients with diabetes.

### How might this impact on clinical practice in the foreseeable future?

- Serum 25-hydroxyvitamin D, calcium and parathyroid hormone levels may not serve as a target for prevention or treatment of coronary artery disease in patients with diabetes, though general population may benefit from calcium-lowering therapy.

## Background

Coronary artery disease (CAD) and related morbidity and mortality are increasing at an alarming rate, in large part, because of increases in aging, obesity, and diabetes [1]. The clinical outcomes associated with CAD are considerably worse for patients with diabetes than for those without diabetes [2]. Therefore, The 2019 European Society of Cardiology (ESC) Guidelines have put a special emphasis on the CAD prevention in patients with diabetes [3].

Calcium (Ca) plays a critical role for almost every aspect in biological processes, such as nerve transmission, enzyme activation, hormone regulation, muscle contraction, and blood clotting [4]. Several observational studies have suggested that serum Ca levels are positively associated with risk of CAD [5, 6]. Vitamin D and parathyroid hormone (PTH) are best known as master regulators of Calcium (Ca) metabolism [7]. Vitamin D motivates the absorption of dietary Ca and PTH mobilizes Ca from the skeleton when serum Ca level is low. Besides, serum 25-hydroxyvitamin D (25OHD) is an important indicator of vitamin D status [8]. Lots of evidence has been accumulated that low level of serum 25OHD levels were associated with cardiovascular risk factors, such as obesity, dyslipidemia and hypertension [9, 10]. In addition, previous study showed that 25OHD may affect endocrine and cardiovascular systems via its nuclear receptor (25-hydroxyvitamin D receptor (VDR)) [11]. As for PTH, previous study suggested that serum PTH levels were associated with cardiovascular mortality in patients with diabetes [12]. Additionally, PTH receptors were found in the endothelial cells, the vascular smooth muscle cells and the cardiomyocytes, which indicated that PTH may serve a role in the pathophysiology of CAD [13, 14]. However, it remains unclear whether serum 25OHD, Ca and PTH levels were causally associated with CAD risk in patients with diabetes.

Mendelian randomization (MR) can avoid some of the limitations of observational studies and is not affected by disease status, thereby avoiding reverse causation bias [15]. Genetic information used to assess associations with explicit outcomes, should be free from confounding [16]. Therefore, genetic variants that influence serum 25OHD, Ca and PTH levels could serve as instrumental variables (IVs) to determine the impact of serum 25OHD, Ca and PTH levels on the risk of CAD in patients with diabetes. We investigated the association between serum 25OHD, Ca and PTH levels and the risk of CAD in patients with diabetes, which has, as far as we know, not been examined previously using the MR approach.

## Methods

### Study design

**Fig. 1:**
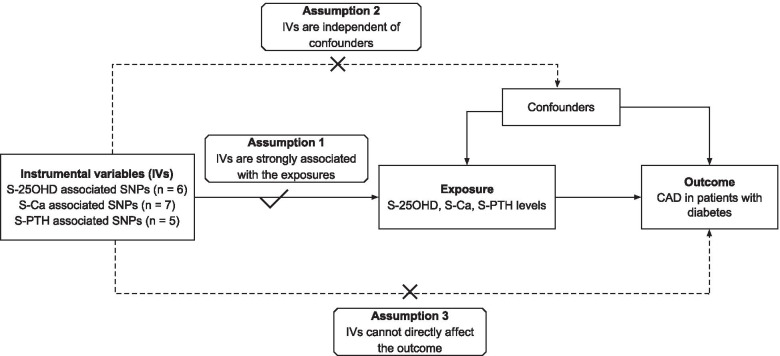
Conceptual schematic of the three main assumptions that underpins the two-sample Mendelian randomization analysis of the association between serum 25-hydroxyvitamin D, calcium, parathyroid hormone levels and the risk of coronary artery disease in patients with diabetes. IV indicates instrumental variables; S-25OHD, serum 25-hydroxyvitamin D; S-Ca, serum calcium; S-PTH, serum parathyroid hormone; CAD, coronary artery disease

A two-sample MR study was designed to appraise whether the genetically determined serum 25OHD, Ca and PTH levels were causally related to the risk of CAD in patients with diabetes. The unbiased relationship between exposures and outcomes could be estimated if the single nucleotide polymorphisms (SNPs) selected as IVs for exposures satisfied the following assumptions: (1) IVs were strongly associated with the exposures concerned; (2) IVs were independent of any possible confounders; (3) IVs must affect outcomes only via the exposures (Fig. 1) [16].

### Data sources

This current two-sample MR study was based on the summary statistics from the recently published genome-wide association study (GWAS). Specifically, genetic signatures associated with serum 25OHD levels were extracted from a large GWAS in 2018, which included 79,366 individuals [17]. The genetic summary data for serum Ca levels were obtained from a large GWAS meta-analysis of 39,400 individuals and < 21,679 additional individuals in 2013 [18]. The genetic association with serum PTH levels were derived from a large GWAS in 2017, with up to 29,155 participants [19]. The summary statistics for CAD in patients with diabetes were obtained from a GWAS in 2018, which consisted of 15,666 individuals with diabetes (3,968 CAD cases, 11,696 controls) from the UK Biobank [20]. All the participants included in present study were of European ancestry.

All the studies that provided data for our analyses have received ethics approvals, and all the participants included in these studies consented to the research. This study only used publicly available data and hence no ethics approval was required.

### SNP selection

To ensure a robust association between IVs and exposures, SNPs associated with serum 25OHD, Ca, PTH levels were selected from corresponding GWAS summary datasets at a genome-wide significant level (*P* < 5 × 10^–8^). In total, six SNPs were identified as IVs for serum 25OHD levels, seven SNPs for serum Ca levels, and five SNPs for serum PTH levels, respectively. For each trait, the selected SNPs were independent and located in different regions, which were validated by the pairwise-linkage disequilibrium test using the LD-Link based on European [21]. Table 1 presented the detail information for the selected SNPs.

**Table 1:** Characteristics of the single-nucleotide polymorphisms associated with serum 25-hydroxyvitamin D, calcium, and parathyroid hormone levels and their associations with coronary artery disease in patients with diabetes

Traits	SNP	Chr	Nearby gene	EA	OA	EAF	25OHD/Calcium/PTH			CAD in patients with diabetes		
							Beta	SE	*P*	Beta	SE	*P*
25OHD	rs3755967	4	GC	C	T	0.72	0.089	0.002	4.74E-343	0.032	0.029	0.274
25OHD	rs12785878	11	DHCR7	T	G	0.75	0.036	0.002	3.80E-62	-0.055	0.033	0.090
25OHD	rs10741657	11	CYP2R1	A	G	0.40	0.031	0.002	2.05E-46	-0.001	0.027	0.972
25OHD	rs17216707	20	CYP24A1	T	C	0.79	0.026	0.003	8.14E-23	-0.033	0.035	0.344
25OHD	rs10745742	12	AMDHD1	T	C	0.40	0.017	0.002	1.88E-14	-0.019	0.028	0.495
25OHD	rs8018720	14	SEC23A	G	C	0.18	0.017	0.003	4.72E-09	0.019	0.034	0.586
Calcium	rs1801725	3	CASR	T	G	0.15	0.071	0.004	8.90E-86	0.052	0.039	0.186
Calcium	rs1570669	20	CYP24A1	G	A	0.34	0.018	0.003	9.10E-12	-0.001	0.028	0.981
Calcium	rs1550532	2	DGKD	C	G	0.31	0.018	0.003	8.20E-11	-0.017	0.028	0.545
Calcium	rs7481584	11	CARS	G	A	0.70	0.018	0.003	1.20E-10	-0.004	0.030	0.904
Calcium	rs780094	2	GCKR	T	C	0.42	0.017	0.003	1.30E-10	0.085	0.028	0.002
Calcium	rs7336933	13	DGKH	G	A	0.85	0.022	0.004	9.10E-10	-0.021	0.037	0.568
Calcium	rs10491003	10	GATA3	T	C	0.09	0.027	0.005	4.80E-09	-0.013	0.046	0.784
PTH	rs6127099	20	CYP24A1	T	A	0.34	0.070	0.003	2.40E-72	0.021	0.030	0.494
PTH	rs4074995	5	RGS14	G	A	0.71	0.030	0.003	3.30E-23	0.029	0.030	0.337
PTH	rs219779	21	CLDN14	G	A	0.75	0.040	0.003	8.90E-22	-0.017	0.030	0.582
PTH	rs4443100	22	RTDR1	G	C	0.32	0.020	0.003	4.10E-11	-0.025	0.029	0.390
PTH	rs73186030	3	CASR	T	C	0.14	0.030	0.004	1.20E-09	0.051	0.039	0.198

*SNP* indicates single-nucleotide polymorphism, *Chr* chromosome, *EA* effect allele, *OA* other allele, *EAF* effect allele frequency, *25OHD* 25-hydroxyvitamin D, *PTH* parathyroid hormone, *CAD* coronary artery disease, *SE* standard error

### Statistical analysis

The conventional inverse-variance-weighted (IVW) method was employed for the major analyses. IVW method used an inverse-variance weighted formula to meta-analyze the ratio estimate of each SNPs, assuming all the SNPs were valid [22]. As a complement, weighted median, MR-Egger, and Mendelian Randomization Pleiotropy Residual Sum and Outlier (MR-PRESSO) methods for MR analyses were conducted in the sensitivity analysis. These methods may provide more robust estimates against pleiotropic instruments. For example, the weighted median method could obtain an unbiased estimate even if up to 50% of the weight came from the invalid SNPs [23]. The MR-Egger and MR-PRESSO methods could detect and correct for pleiotropy and outliers, respectively [24, 25].

Heterogeneity between SNPs in the IVW analysis was estimated by Q statistics and visually inspected by funnel plots (Figure S1-3) [23, 26]. Potential directional pleiotropy was estimated by the MR-Egger intercept test [24]. *P* < 0.05 indicated the present of heterogeneity and pleiotropy, respectively. In addition, a leave-one-out analysis was performed, in which one exposure-associated SNP was removed at a time to identify any pleiotropic or outlying SNP. Furthermore, the PhenoScanner V2 was searched to detect any potential pleiotropic associations between selected SNPs and confounders [27]. The statistical power was calculated on mRnd (https://shiny.cnsgenomics.com/mRnd/) [28]. Based on the sample size of 15,666 and 0.46% of variance in 25OHD, 0.21% of variance in Ca, and 0.36% of variance in PTH explained by the selected SNPs, our MR analyses had 80% power at an alpha rate of 5% to detect an OR of 1.88 per SD unit of 25OHD, 2.35 per SD unit of Ca, 2.01 per SD unit of PTH.

A *P* value < 0.017 was deemed statistically significant after the Bonferroni correction for three exposures. All the statistical analyses were performed in RStudio (R version 3.6.2).

## Results

Six SNPs likely to genetically determine the serum 25OHD levels were identified as IVs for serum 25OHD levels, seven SNPs for serum Ca levels, and five SNPs for serum PTH levels, respectively. Table 1 presented the genetic associations between IVs for serum 25OHD, Ca, and PTH levels and the risk of CAD in patients with diabetes. After searching in the PhenoScanner, rs780094 was found strongly associated with metabolic traits such as blood glucose, triglycerides, and total cholesterol, which may represent a pleiotropic SNP, while the remained SNPs were not related to any traditional risk factor of CAD in patients with diabetes.

### The effect of serum 25OHD levels on the risk of CAD in patients with diabetes

**Fig. 2:**
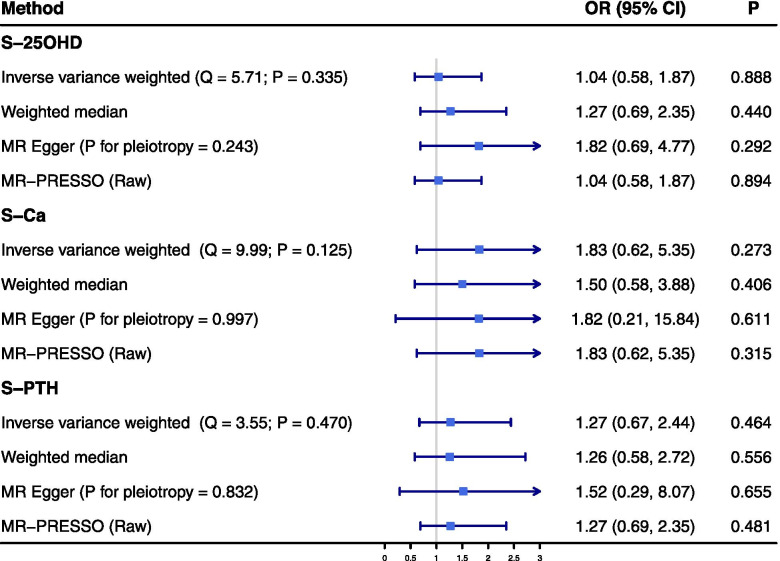
Summary Mendelian randomization estimates of the associations between serum 25-hydroxyvitamin D, calcium, parathyroid hormone levels and the risk of coronary artery disease in patients with diabetes. OR indicates odds ratio; CI, confidence interval; IVW, inverse variance-weighted; MR, Mendelian randomization; MR-PRESSO, MR Pleiotropy Residual Sum and Outlier; S-25OHD, serum 25-hydroxyvitamin D; S-Ca, serum calcium; S-PTH, serum parathyroid hormone

There was no significant association between genetically predicted serum 25OHD levels, based on six SNPs, and the risk of CAD in patients with diabetes in the IVW analysis (odds ratio (OR) = 1.04, 95% confidence interval (CI): 0.58 - 1.87, *P* = 0.888). In line with this primary result, no significant association was observed in the sensitivity analyses using the weighted median, MR-Egger, and MR-PRESSO methods (Fig. 2). No evidence of heterogeneity was observed between the estimates of selected six SNPs (Q = 5.71, *P* = 0.336), and no potential directional pleiotropy was revealed in the MR-Egger intercept test (*P* = 0.243). Figure S4 presented the results of leave-one-out analysis, which indicated that rs3755967 might affect the association between serum 25OHD levels and the risk of CAD in patients with diabetes (Overall OR = 1.04, *P* = 0.888; OR after removing rs3755967 = 0.47, *P* = 0.149). However, rs375596 was not related to any traditional risk factor of CAD in patients with diabetes, based on the Phenoscanner search, namely, no evidence to support that rs375596 was a potential pleiotropic SNP was observed.

### The effect of serum Ca levels on the risk of CAD in patients with diabetes

No significant association between genetic predisposition to serum Ca levels and the risk of CAD in patients with diabetes was found in the IVW analysis (OR = 1.83, 95% CI: 0.62 – 5.35, *P* = 0.273). Similar results were observed in the sensitivity analyses (Fig. 2). Q statistics suggested no evidence of heterogeneity (Q = 9.99, *P* = 0.125) and the intercept of MR-Egger regression indicated no evidence of pleiotropy (*P* = 0.997). Though rs780094 was a potential pleiotropic SNP based on Phenoscanner search, the result of leave-one-out analysis indicated that association was attenuated but still insignificant (Overall OR = 1.83, *P* = 0.273; OR after removing rs780094 = 1.32, *P* = 0.529) (Figure S5).

### The effect of serum PTH levels on the risk of CAD in patients with diabetes

The IVW analysis suggested no significant association between genetically determined serum PTH levels and the risk of CAD in patients with diabetes (OR = 1.27, 95% CI: 0.67 – 2.44, *P* = 0.464), which was confirmed by the sensitivity analyses using the weighted median, MR-Egger and MR-PRESSO methods (Fig. 2). In addition, no evidence of heterogeneity (Q = 3.55, *P* = 0.470) and pleiotropy (*P* = 0.832) was observed in our study. The leave-one-out analysis indicated that this insignificant association was not disproportionally driven by any individual SNP (Figure S6).

## Discussion

To the best of our knowledge, this is the first two-sample MR study to explore the association of serum 25OHD, Ca and PTH levels with the risk of CAD in patients with diabetes. The results of this MR study showed that no evidence approved causal effects of genetically determined serum 25OHD, Ca and PTH levels on CAD risk in patients with diabetes. The findings were robust in sensitivity analyses with different statistical models.

Evidence from previous multiple studies indicates that increased serum Ca, a short term consequence of Ca supplementation, is associated with an increased risk and mortality of CAD [29]. Several traditional observational studies individuals with a subsequent elevation of serum Ca levels showed increased extent of coronary artery calcification [30, 31], increased incidence of stroke [32], increased risk of developing heart failure [33], and an increased risk of cardiovascular mortality [34]. Possible mechanisms whereby elevated serum Ca levels may increase the risk of CAD include effects on calcification, blood coagulation, and altered gene expression induced by effects on arterial wall Ca-sensing receptor [35].

25-hydroxyvitamin D is the major circulating form of vitamin D and is used as indicator of vitamin D status [36]. As critical regulators for Ca metabolism, 25OHD can exert a biological effect mainly through regulating intracellular and extracellular Ca concentrations [7]. In addition to the regulation of Ca metabolism, 25OHD is involved in cell proliferation and differentiation and exerts an immunomodulatory and anti-inflammatory property. Evidence from previous studies revealed that 25OHD deficiency resulted in the increase expression of inflammatory factors, such as interleukin 6, interleukin 8, and C-reactive protein [37], which play important roles in CAD. Furthermore, low serum 25OHD levels are associated with obesity, dyslipidemia, the metabolic syndrome, and hypertension, which are all well-established CAD risk factors [9, 10]. It was shown that lower serum bioavailable and free 25OHD levels were associated with prevalent cardiovascular mortality independent of other recognized cardiovascular risk factors [38]. Vitamin D deficiency might be considered a so-called “nonclassical” CAD risk factor in studies without a specific focus on diabetes [39, 40]. However, the two recent large scale RCTs failed to demonstrate a beneficial effect of 25OHD on CAD [41, 42].

Consistently, PTH is a key regulator of Ca homeostasis. With further in-depth studies, PTH appears to play a role in the cardiovascular system [13]. A number of epidemiologic studies showed associations of mild hyperparathyroidism with hypertension, endothelial dysfunction and metabolic syndrome, which were associated to an increased incidence of CAD [13]. A recent observational cohort study showed a significant association between PTH levels and cardiovascular mortality in patients with diabetes, which was true after adjustment for classical CAD risk factors [12]. However, a systematic review and meta-analysis of prospective studies suggested no significant association between serum PTH levels and CAD [43].

The association of serum 25OHD, Ca and PTH levels with the risk of CAD is a controversial issue, and the findings seem to vary depending on the investigated population. Our study focuses on a population with diabetes. Until then, very little research has been done in this area. We subsequently performed a MR study to explore the association of serum 25OHD, Ca and PTH levels with the risk of CAD in patients with diabetes. To date, randomized controlled trials (RCTs) remain the gold standard to establish causal relationships [44]. However, RCTs cannot always be conducted, because they can be excessively costly and require a long period to demonstrate the effect on CAD events [15]. When the number of confounders is too large, or when confounders are uncertain, the observed associations may not reflect the causality but may arise as the result of confounding or reverse causation [45, 46]. In the absence of high-quality RCTs and given the inconclusive results from the existing observational studies, MR may serve as a time- and cost-efficient alternative approach and contribute to its increasing popularity for assessing and screening for potentially causal associations [15, 47]. A recent study without a specific focus on diabetes has used this technique to demonstrate that a genetic predisposition to higher serum Ca levels was associated with increased risk of CAD and myocardial infarction [48]. In a large, well-powered MR study, genetically lowered serum 25OHD levels were not associated with increased risk of CAD [49]. Melhus et al. used the MR approach to demonstrate that genetic predisposition to higher serum PTH concentrations did not appear to be an independent risk factor for CAD among the general population [50].

Consistent with the results in the general population [49, 50], we found no evidence to support the causal association of serum 25OHD and PTH levels with the risk of CAD in patients with diabetes with the aid of the hitherto largest GWAS meta-analysis. Unlike previous studies [48, 51], our results provided evidence against a causal role for elevated serum Ca levels in CAD susceptibility, because of our focus on patients with diabetes. Serum 25OHD, Ca, and PTH levels should therefore not be regarded as independent risk factors for CAD in patients with diabetes. Those associations reported in the past might be due to indirect effects by residual confounding. Serum 25OHD, Ca and PTH levels are more likely biomarkers rather than causal risk factors for CAD in patients with diabetes.

The major strength of this study is the MR design, using 25OHD-, Ca-, and PTH-related SNPs and SNPs-CAD in patients with diabetes from a recently published large-scale GWAS, which minimized reverse causation bias and confounding that can produce false associations in traditional observational studies. Using data from a large genetic consortium for 25OHD (*n* = 79,366), serum Ca (*n* =39,400), PTH (*n* = 29,155) levels and CAD risk (3,968 cases and 11,696 controls) in patients with diabetes has enabled us to more precisely test our study hypothesis than if we had used individual-level data from a small study. Furthermore, in the absence of large-scale, long-term RCT data, our findings provide strong evidence against a causal role for low serum 25OHD levels, elevated serum Ca and PTH levels in CAD susceptibility in patients with diabetes.

Our study also has several potential limitations that deserve discussion. First, potential pleiotropy is difficult to exclude in any MR study. However, all the results analyzed with other more robust methods in the sensitivity analyses were consistent with the primary results. The null result could also be explained by canalization, which is defined as compensatory feedback interactions [52]. Second, we only investigated explore the association of serum 25OHD, Ca and PTH levels with the risk of CAD in patients with diabetes from a genetic point of view. We were unable to examine whether there are any interactions between genetically predicted serum 25OHD, Ca and PTH levels and lifestyle/environmental factors on the risk of CAD in patients with diabetes. Third, the examined GWAS were primarily conducted in individuals of European ancestry, which confines the transferability of the present findings to other ethnicities. Thus, studies in a causal association are warranted to verify the present conclusions among individuals of different ancestries. Finally, our results did not reach a statistical power of 80%, which probably caused by the low variance in exposures explained by the selected SNPs and the insufficient sample size. Therefore, it should be cautious to make the conclusion and we had to admit that there was a possibility that false-negative results may occur.

## Conclusions

Overall, serum 25OHD, Ca, and PTH levels may not be causally associated with the risk of CAD in patients with diabetes. Serum 25OHD, Ca and PTH levels may not serve as a target for prevention or treatment of CAD in patients with diabetes. Further investigations are warranted to validate our findings.

## Availability of supporting data

The datasets analyzed in this study are publicly available summary statistics.

## Supplementary Information

**Additional file 1: Figure S1.** Funnel plot of the Mendelian randomization estimate for the association between serum 25-hydroxyvitamin D levels and the risk of CAD in patients with diabetes. **Figure S2.** Funnel plot of the Mendelian randomization estimate for the association between serum calcium levels and the risk of CAD in patients with diabetes. **Figure S3.** Funnel plot of the Mendelian randomization estimate for the association between serum parathyroid hormone levels and the risk of CAD in patients with diabetes. **Figure S4.** Leave-one-out analysis of the association between serum 25-hydroxyvitamin D levels and the risk of coronary artery disease in patients with diabetes. **Figure S5.** Leave-one-out analysis of the association between serum calcium levels and the risk of coronary artery disease in patients with diabetes. **Figure S6.** Leave-one-out analysis of the association between serum parathyroid hormone levels and the risk of coronary artery disease in patients with diabetes.

## Abbreviations

Ca: Calcium
CAD: Coronary artery disease
ESC: European Society of Cardiology
GWAS: Genome-wide association study
IV: Instrumental variables
IVW: Inverse-variance-weighted
MR: Mendelian randomization
MR-PRESSO: Mendelian Randomization Pleiotropy Residual Sum and Outlier
PTH: Parathyroid hormone
VDR: 25-hydroxyvitamin D receptor
25OHD: 25-hydroxyvitamin D
