# Supplementary material for: The impact of serum 25-hydroxyvitamin D, calcium, and parathyroid hormone levels on the risk of coronary artery disease in patients with diabetes: a Mendelian randomization study
Source: Nutr J. 2021 Oct 3;20:82. doi: 10.1186/s12937-021-00735-z (PMC8489084; doi:10.1186/s12937-021-00735-z)
Supplement: Supplementary file 1 — Additional file 1: Figure S1. Funnel plot of the Mendelian randomization estimate for the association between serum 25-hydroxyvitamin D levels and the risk of CAD in patients with diabetes. Figure S2. Funnel plot of the Mendelian randomization estimate for the association between serum calcium levels and the risk of CAD in patients with diabetes. Figure S3. Funnel plot of the Mendelian randomization estimate for the association between serum parathyroid hormone levels and the risk of CAD in patients with diabetes. Figure S4. Leave-one-out analysis of the association between serum 25-hydroxyvitamin D levels and the risk of coronary artery disease in patients with diabetes. Figure S5. Leave-one-out analysis of the association between serum calcium levels and the risk of coronary artery disease in patients with diabetes. Figure S6. Leave-one-out analysis of the association between serum parathyroid hormone levels and the risk of coronary artery disease in patients with diabetes. [file 12937_2021_735_MOESM1_ESM.docx]

**Supplementary Materials**

Figure S1. Funnel plot of the Mendelian randomization estimate for the association between serum 25-hydroxyvitamin D levels and the risk of CAD in patients with diabetes.

Figure S2. Funnel plot of the Mendelian randomization estimate for the association between serum calcium levels and the risk of CAD in patients with diabetes.

Figure S3. Funnel plot of the Mendelian randomization estimate for the association between serum parathyroid hormone levels and the risk of CAD in patients with diabetes.

Figure S4. Leave-one-out analysis of the association between serum 25-hydroxyvitamin D levels and the risk of coronary artery disease in patients with diabetes.

Figure S5. Leave-one-out analysis of the association between serum calcium levels and the risk of coronary artery disease in patients with diabetes.

Figure S6. Leave-one-out analysis of the association between serum parathyroid hormone levels and the risk of coronary artery disease in patients with diabetes.

Figure S1.

Figure S2.

Figure S3.

Figure S4.

Figure S5.

Figure S6.
